# Teaching Emergency Medical Technicians about Advanced Life Support Interventions: Pilot Study of an Online Continuing Education Course

**DOI:** 10.1017/dmp.2025.10171

**Published:** 2025-08-11

**Authors:** Enzo G. Plaitano, Bianca L. Pate, Kevin M. Ryan

**Affiliations:** 1 Boston University EMS, Boston, MA, USA; 2 Department of Emergency Medicine, Boston University Chobanian & Avedisian School of Medicine, Boston, MA, USA; 3 Washington & Jefferson College EMS, Washington, PA, USA

**Keywords:** emergency medical services, continuing education, emergency medical technician, ALS assist skills, BLS-ALS interface

## Abstract

**Objectives:**

Emergency Medical Technician (EMT) scope of practice guidelines in the US suggest that EMTs should assist paramedics with advanced skills during patient care. However, learning to assist with these skills is not an EMT national education requirement. This study examined the feasibility and impact of a short, online pilot continuing education course in providing EMTs with the confidence and basic knowledge to assist with advanced interventions.

**Methods:**

The pilot cohort included licensed EMTs (*n*=10) self-enrolled in a continuing education class listed on the institution’s EMS continuing education website and advertised on social media. Optional, anonymous questionnaires and multiple-choice exams were administered to students pre/post-course. Statistical analysis included paired nonparametric tests.

**Results:**

Total scores were 43% higher on the post-exam (88/100, 95% CI [76, 100]) compared to the pre-exam (45/100, 95% CI [37, 53]) (*P*<0.05). Self-reported comfort was higher on the post-evaluation for needle thoracostomy (95% increase), advanced airways (25% increase), EKGs (19% increase), intravenous access (14% increase), and communication (22% increase).

**Conclusions:**

Results suggest that short, online continuing education courses on BLS-ALS interface for EMTs might be efficacious in improving both comfort and knowledge of selected advanced interventions often used by paramedics, although larger future studies are needed.

Almost 70% of licensed emergency medical services (EMS) clinicians in the US are certified as an Emergency Medical Technician (EMT) or below and limited to delivering Basic Life Support (BLS). Because paramedics only make up 25% of the workforce,^1^ EMTs often work with paramedics to provide advanced care to critically sick or injured patients.^2^ This Advanced Life Support (ALS) care includes definitive airway management, intravenous and intraosseous therapy, and cardiac monitoring.^2^ Studies suggest that paramedic/EMT teams can function just as well as dual paramedics; however, these combined teams can also have longer on-scene times.^3^

While directly performing ALS skills is not included in the EMT scope of practice, paramedics may ask for assistance in preparing the necessary supplies. The National EMS Scope of Practice Model states that EMTs are expected to “assist higher-level personnel at the scene and during patient transport,” but this education is not required by the National EMS Education Standards.^2^ This provides a significant gap between the protocol expectations and clinical abilities of EMTs.

A recent “Advanced Medical Life Support - Basics” national course was designed to cover these advanced interventions at a level appropriate for EMTs.^4^ However, the course is 16 hours, which is a barrier for completion.^4^ Others have created shorter, 3-hour education sessions, and found that EMTs have moderate success rates with performing select ALS interventions.^5^ However, that course aimed to determine if EMTs could perform some skills independently for future protocols, but not assist with interventions that are already part of clinical practice. Since EMTs must complete continuing education every 2 years to remain nationally certified, these short continuing education courses can be used to bridge this gap between expectations and practice.^6^ While several online BLS-ALS interface courses do exist, there has been little formal evaluation on the effectiveness of these courses in improving the confidence and knowledge of EMTs.^7,8,9^

To address this gap, our academic institution designed a 3-hour, online continuing education course on BLS-ALS interface. The course included topics similar to other online courses (assisting with IV administration, EKG placement, advanced airway management, medication administration).^7,8,9^ Therefore, our objective was to determine the impact of a single online course in providing EMTs with the comfort and basic knowledge of assisting with ALS interventions.

## Methods

### Ethical Approval and Data Sources

The Boston University Institutional Review Board approved the data collection methods in this study (H-40458). After providing electronic informed consent, data collection was performed in compliance with anonymous survey guidelines through Qualtrics (Qualtrics, Provo, UT, USA).

### Course Description

The Boston University Division of EMS Education hosted an online continuing education course titled “BLS-ALS Interface: How to Better Assist the ALS Provider” in February 2021. The single, 3-hour course was approved by the Massachusetts Office of EMS and taught by an EMS Instructor (Supplemental 1). EMTs self-enrolled in the course for credits through the continuing education website. The curriculum included intravenous (IV) and intraosseous (IO) access, electrocardiograms (EKGs), needle thoracostomy, and advanced airways. Information to develop this course was gathered from the Paramedic National Curriculum, literature highlighting best ALS practices, and our institutional experience in BLS-ALS interactions within our EMS agency.^2,6^ We ensured that the content aligned with other online BLS-ALS interface courses.^7,8,9^ The course was taught live through an electronic platform (Zoom, Santa Clara, CA, USA). First, it included didactic lectures focused on the indications, contraindications, pathophysiology, and equipment utilized during each intervention (Supplemental 1). Then, instructional skills videos demonstrated the ALS skills, which were publicly available through YouTube (Google, San Bruno, CA, USA) (Supplemental 2). Lastly, students were sent into breakout rooms to apply application-based group scenarios with various medical and trauma patients, where they discussed ALS interventions and equipment preparation with each other (Supplemental 3).

### Cohort Description

The cohort included Massachusetts state licensed EMT-Basics (EMTs) who self-enrolled in an online continuing education class at Boston University EMS. The course was listed on the institution’s EMS continuing education website^10^ and advertised briefly on its social media. At the time of the course, Boston University EMS had 56 affiliated EMTs and 11 (20%) registered to take this optional continuing education course. Participants had to pay a small fee to enroll.

### Questionnaires

The surveys were constructed and validated by EMS Instructors at our institution as well as the Institutional Review Board for cultural appropriateness, accuracy for this target population, and general comprehension of the questions. After this review and a cognitive debrief with EMS clinicians who tested the survey, content and phrasing were adjusted as appropriate. Students were administered these electronic questionnaires pre/post-course. The pre-course survey included demographics, self-reported 5-point Likert scales regarding comfort to assist with the ALS skills, and a 10-question multiple-choice exam on ALS interventions. The post-course survey was administered immediately upon completion of the program and included these same measures. Students did not receive their exam scores or any feedback on the pre-course exam before the post-exam. During exams, students did not have access to course materials and were monitored through video to ensure an individual effort. Each question included a scenario and 4 choices; 1 choice had the correct intervention, equipment, or landmark (Supplemental 4).

### Statistical Analysis

Statistical analysis was performed with JMP Pro 17 (SAS Institute, Cary, NC, USA). We only performed statistical significance testing on our main outcome of pre/post-course total exam scores (0%-100% correct) using paired Wilcoxon signed-rank tests, which was selected due to the ordinal data and resilience to small samples. Due to this small pilot sample, we chose to only examine magnitude of effects instead of significance values for all other variables. Significance tests are often flawed in small samples and can lead to false negative results (“Type II errors”).^11^

## Results

### Participant Demographics

While 11 students completed the course, 1 student did not provide consent for study participation. The study cohort therefore included 10 students (participation = 91%), who had a 100% response rate on the pre/post-evaluations. Students completed all survey questions so there was no missing data. The students were mostly female (70%) and had a median of 4.0 (IQR 2.0-5.75) years of experience as an EMT. Only 4 EMTs (40%) felt comfortable working as part of an ALS unit after completing their initial EMT course and only 3 (30%) stated that their previous education prepared them to assist with ALS interventions. Additionally, only 1 student (10%) previously attended a course on ALS-BLS interface. All 4 students (100%) who reported working with paramedics regularly indicated that they have been asked to assist paramedics with advanced interventions.

### Cognitive Exam Performance

Total exam scores were 43% higher, on average, on the post-exam (mean = 88, 95% CI [76, 100]) compared to the pre-exam (mean = 45, 95% CI [37, 53]) (*P*<0.05) (Table 1). Half of the EMTs scored 100% on the post-exam, while the highest score was 60% on the pre-exam. Regarding vascular access, correct answers were higher on the post-exam for IV drip sets (90% higher), intraosseous needle length (70% higher), IV catheter size (40% higher), and IV start supplies (40% higher). Regarding airway management, correct answers were higher on the post-exam for intubation laryngoscope blade size (60% higher) and location for needle thoracostomy (50% higher), but not for selecting the most definitive airway device (0% increase). Regarding cardiac monitoring, correct answers were higher on the post-exam for location for lead V2 placement (60% higher), number of electrodes for a 12-lead EKG (30% higher), and correctly identifying a ST Elevation Myocardial Infarction (STEMI) (20% higher).

### Self-reported Comfort with ALS Skills

Self-reported comfort in assisting paramedics with interventions was higher on the post-course survey when compared to the pre-course survey for all topic areas, including needle thoracostomy (95% increase), advanced airways (25% increase), 12-lead EKGs (19% increase), IV access (14% increase), and communicating with ALS providers (22% increase) (Table 2).

## Limitations

This pilot study was limited by a small sample size of only 10 EMTs that enrolled in this optional continuing education class. Average enrollment across all courses ranged from 8-12 students during that academic year. Due to this small sample, we primarily examined effect sizes instead of significance tests to help avoid making false claims of no significance (“Type II errors”).^11^ Therefore, future studies in larger samples should examine the significance of all effect sizes.

Generalizability of these results was also limited, as this sample only included collegiate EMTs from a single institution in Boston. EMT scope of practice and protocols vary widely between states, and some of the skills within these programs are already incorporated into other state EMT protocols. Therefore, these study results may not be generalizable to EMTs nationally.

Additionally, there is the potential for response bias given the self-reported nature of the survey questions. However, students were told that the surveys were anonymous and that they received no contributions or retributions for responses. Students were told that they would receive the continuing education credit regardless of their responses or if they did not complete the surveys.

Cheating on the cognitive exam was also possible. Students were advised of the anonymous results and monitored through video which may have diminished cheating behaviors. While cheating may have been limited, the phenomenon of learning effects inherent in pre/post studies could be possible, as some degree of increased scores could be due to the students’ familiarity.

Lastly, we did not examine if this education translates to improved team-based decision making or patient outcomes in the prehospital setting. The potential for this longitudinal follow-up was limited in this study, as only 4 EMTs endorsed regularly working with paramedics. Future studies could include more providers who interface with paramedics and examine long-term outcomes.

## Discussion

Most EMS clinicians in the United States are licensed as EMTs and approximately 23% of EMS agencies are staffed solely with BLS providers.^12^ Therefore, EMTs must often work with paramedics to care for critically sick or injured patients. While knowledge of ALS skills is not mandated for EMTs nationally,^2^ numerous state protocols highlight “ALS-assist” skills.^14^ Although several online continuing education courses have been developed to bridge this gap in practice,^7,8,9^ studies on the effectiveness of these online BLS-ALS courses are extremely limited.

In this small pilot study, only 30% of EMTs reported that their initial EMT certification course prepared them to work alongside paramedics and only 10% reported attending a prior BLS-ALS class. On the post-course evaluations, EMTs reported increased comfort and demonstrated higher knowledge of ALS skills. This could increase teamwork between EMTs and paramedics and help reduce on-scene times^3^ and risky medical errors during unpredictable emergency care settings.^13^

Previous studies demonstrate that EMTs can learn to place supraglottic airways and gain intraosseous access following a 3-hour course on these ALS interventions.^5^ Our study builds on this work by examining if EMTs can learn about a wider range of ALS skills (Table 1). Overall, EMTs had higher total post-exam scores and higher post-exam scores on all individual questions besides choosing the most definitive airway. However, 80% of the EMTs correctly selected endotracheal intubation on the pre-exam, which suggests that advanced airway management is already well conveyed in traditional EMT education. Pre-exam scores were also high for cardiac monitoring items, as many states, including Massachusetts, already allow EMTs to acquire and transmit 12-lead EKGs.^14^ However, results suggest that knowledge of IV skills was low on the pre-exam and had the largest increase in post-exam scores. Given the commonality of this skill, IV topics should remain a focus of future BLS-ALS courses.^7,8,9^ In contrast, EMTs self-reported the largest increase in comfort post-course for needle decompression skills (Table 2). Given that only 10% of EMTs reported any comfort pre-course, this skill should continue to be emphasized.

## Conclusions

Our results suggest that short, online continuing education courses on BLS-ALS interface for EMTs^7,8,9^ might be efficacious in improving both comfort and knowledge of selected advanced interventions often used by paramedics. While this pilot study only included a small cohort of EMTs and future research is needed with a larger sample, our results suggest that an online continuing education course on BLS-ALS interface is feasible for providing education to EMTs.

## Supplementary Material

Supplementary Material

The supplementary material for this article can be found at http://doi.org/10.1017/dmp.2025.10171.

## Figures and Tables

**Table 1.** Cognitive exam results before and after the continuing education course

	Pre-course percent correct	Post-course percent correct	Differences in percentages
Exam topic	Mean (95% CI)	Mean (95% CI)	(Post - Pre)
**Overall exam score**	45 (37, 53)	88 (76, 100)	43

**Vascular access**			

IV tubing drip rate	10 (0, 33)	100 (100, 100)	90

IO needle length	20 (0, 50)	80 (50, 100)	60

IV catheter size	60 (23, 97)	100 (100, 100)	40

IV access supplies	40 (3, 77)	80 (50, 100)	40

**Airway management**			

Laryngoscope blade size	30 (0, 65)	90 (67, 100)	60

Needle thoracostomy location	30 (0, 65)	80 (50, 100)	50

Most definitive airway device	80 (50, 100)	80 (50, 100)	0

**Cardiac monitoring**			

Lead V2 placement location	40 (3, 77)	100 (100, 100)	60

Number of 12-lead electrodes	60 (23, 97)	90 (67, 100)	30

STEMI identification	60 (23, 97)	80 (50, 100)	20

*Note:* Mean exam percentages ranged from 0-100% correct with 10 total questions in the exam.

**Table 2.** Self-reported comfort before and after the continuing education course

	Pre-course comfort	Post-course comfort	
Topic	Mean (95% CI)	Mean (95% CI)	% Increase
Needle thoracostomy	2.0 (1.4, 2.6)	3.9 (3.3, 4.5)	95

Advanced airways	3.2 (2.6, 3.8)	4.0 (3.5, 4.5)	25

12-lead EKGs	3.6 (2.8, 4.4)	4.3 (3.8, 4.8)	19

Intravenous access	3.6 (2.8, 4.4)	4.1 (3.6, 4.6)	14

Communication	3.2 (2.6, 3.8)	3.9 (3.2, 4.6)	22

*Note:* Self-Reported comfort was measured on a Likert scale from 1–5 with higher scores indicating higher comfort. Percent increase is the change from pre-course to post-course scores.
