# Supplementary material for: Teaching Emergency Medical Technicians about Advanced Life Support Interventions: Pilot Study of an Online Continuing Education Course
Source: Disaster Med Public Health Prep. Author manuscript; Available in PMC 2025 Aug 18. (PMC12359697; doi:10.1017/dmp.2025.10171)
Supplement: Supplementary Material [file NIHMS2104426-supplement-Supplementary_Material.docx]

**
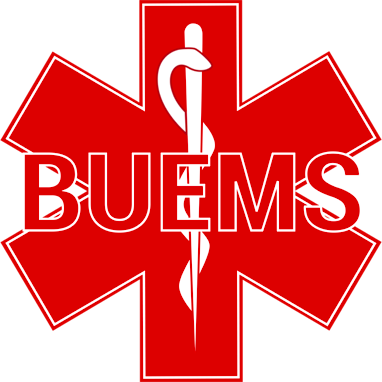
SUPPLEMENTAL 1: COURSE OUTLINE**

**BLS-ALS Interface**

February 17th, 2021 (3 hours)

Continuing Education Course

Boston University Emergency Medical Services

**Course Description:** This course is designed to provide BLS providers with the knowledge to feel more comfortable in the ALS setting and to support the ALS provider by becoming a more skilled team member. This course will build upon your BLS experience and provide exciting new information and skills regarding EKGs (3-lead, 5-lead, 12-lead), oral intubation and other advanced airways, needle decompression, intravenous (IV) access, intraosseous (IO) access, and EtCO2 monitoring, among others. The course will emphasize the importance of basic life support to patient survival, the integration of effective basic life support with advanced life support interventions, and the importance of effective team interaction and communication during patient care. This course does not expand your current scope of practice nor does it certify you as an ALS provider. The goal is to supply BLS providers with advanced knowledge and skills to better assist the ALS provider. If time permits, this course will also cover new and exciting prehospital critical care interventions, such as field extracorporeal membrane oxygenation (ECMO), amputations, and finger thoracostomies.

**Course Format:** This course will be taught electronically through the platform, *Zoom*. The course will include learning through instructor lectures, photograph descriptions, infographics, research study findings, YouTube videos, instructor-led self-simulations, and break-out room discussions.

**Part 1: Background of EMS Providers and Team Dynamics**

(Approx. 20 minutes)

**Topic 1: Introduction / Course Title**

*Quick Overview of Course Outline*

**Topic 2: About the Instructor**

*Clinical and EMS Research Background; Contact Information*

**Topic 3: Disclaimer**

*Discuss that this course does not expand your current scope of practice nor does it certify you as an ALS provider*

**Topic 4: BLS Overview**

*BLS Protocols, Skills, Scope of Practice*

**Topic 5: ALS Overview**

*ALS Protocols, Skills, Scope of Practice*

**Topic 6: Types of ALS Providers (Not Available in All States)**

*Advanced-EMT; Paramedic; PHRN; Physician Extender; Prehospital Physician*

**Topic 7: Types of ALS Responses**

*Full Medic Unit, Fly Car, Firefighter ALS Provider; Off-duty provider*

**Part 2: Actions of Requesting ALS** (Approx. 20 minutes)

**Topic 8:** **When Should You Request ALS?**

*Criteria and Tips/Advice Regarding Requesting ALS Assistance*

**Topic 9: What Can ALS Do That I Can’t Do**

*More In-depth Overview of the Patients, Scenarios, Diseases/injuries that would Benefit from ALS Assistance*

**Topic 10:** **I Requested ALS...Now What Do I Do Before They Arrive?**

*Discuss How to Prepare for ALS Arrival and What BLS Providers Can do in the Interim to ALS Arrival*

**Topic 11:** **Effective Team Interaction and Communication During Patient Care - Two-Way Street!**

*Discuss How to Interact and Communicate with ALS, Along with Team Dynamics*

*Built-In 5 Minute Break (Restroom, Water, etc.)*

**Part 3: ALS Skills and How to Assist the ALS Provider Regarding these Skills** (Approx. 1 hour 30 minutes)

*For each topic below, we will explore:*

**Physiology** - Why do the intervention

**Terminology** - What each piece of equipment is called and what each piece does

**Functionality** - How intervention works

**Outcome** - Final outcome of intervention

**Complications and Risks** - Of intervention

**Review of how you can help** - YOUR tasks during these skills

*These topics will include a mix of:*

1. Instructor lecture with inserted photos for explanation
2. Instructor-led simulation with students finding landmarks and performing simulated interventions on themselves (i.e. location of electrode placement, decompression location, finding veins and bones for IV and IO access, etc.),
3. YouTube Videos to show complete interventions

**Topic 12:** EKGs (3-lead, 5-lead, 12-lead)

**Topic 13:** Oral intubation

**Topic 14:** Other advanced airways (Supraglottic airways)

**Topic 15:** Chest Needle Decompression

**Topic 16:** Intravenous (IV) Access

**Topic 17:** Intraosseous (IO) Access

**Topic 18:** EtCO2 Monitoring

**Topic 19:** Aeromedical Crew Assistance and Aircraft Safety

**Topic 20:** Overview of Interventions with Main Takeaways

*Built-In 5 Minute Break (Restroom, Water, etc.)*

**Part 4: Group Scenarios (Breakout Rooms) and Discussion Regarding BLS-ALS Interface**

(Approx. 30 minutes)

*For each scenario below, students will explore and discuss:*

1. What should the BLS providers do before ALS arrival?
2. What should BLS and ALS providers do upon ALS arrival?
3. How can the BLS provider assist the ALS provider with skills/interventions?

**Topic 21:** Example Scenario for Students (performed by instructor)

**Topic 22:** Scenario #1 Trauma Patient

**Topic 23:** Scenario #2 Medical Patient

**Topic 24:** Scenario #3 Multiple Patients

**Part 5: Special ALS Topics New To EMS** (Approx. 10 minutes)

**Topic 25:** Field Extracorporeal Membrane Oxygenation (ECMO)

**Topic 26:** Surgical Field Amputations

**Topic 27:** Finger Thoracostomies

**SUPPLEMENTAL 2: YOUTUBE REFERENCES USED IN COURSE**

| ALS topics | Link | Citation |
| --- | --- | --- |
| 12 Lead EKG |  |  |
| 12 lead EKG placement | https://youtu.be/0gAOy7f2-Gs | Theriault, R. 12 Lead ECG Placement Example [Video]. YouTube. <https://youtu.be/0gAOy7f2-Gs>. Published February 18, 2015. |
| Airway Management | | |
| ET intubation setup and technique | https://youtu.be/-M3OlfmKjO8 | DeClerck, M. Intubation Procedure Setup and Technique [Video]. YouTube. <https://youtu.be/-M3OlfmKjO8>. Published April 3, 2020. |
| ET intubation using video laryngoscope | https://youtu.be/WAxPTBNGX3g | Dilon Technologies Inc. Video Laryngoscope Stylet Intubation [Video]. YouTube. <https://youtu.be/WAxPTBNGX3g>. Published March 17, 2014. |
| King airway | https://youtu.be/yJGuxDny_fo | Mason, J. King LT Supraglottic Airway [Video]. YouTube. <https://youtu.be/yJGuxDny_fo>. Published March 15, 2016. |
| LMA airway | https://youtu.be/zXgvTWf7gdU | Mason, J. LMA Supraglottic Airway [Video]. YouTube. <https://youtu.be/zXgvTWf7gdU>. Published March 15, 2016. |
| Needle Decompression | | |
| Needle decompression | https://youtu.be/DN9ewqnoiow | North American Rescue. SPEAR Needle Decompression- Left Anterior [Video]. YouTube. https://youtu.be/DN9ewqnoiow. Published November 9, 2018. |
| IV Access |  |  |
| Priming an IV bag | https://youtu.be/4ntqS_R1r70 | RegisteredNurseRN. How to Prime IV Tubing Line \| How to Spike a IV Bag for Nursing [Video]. YouTube. https://youtu.be/4ntqS_R1r70. Published March 10, 2017. |
| Placing an IV catheter | https://youtu.be/OWZfCYze-AQ | Paramedic Learning. Starting a 20G Antecubital/Cephalic Vein IV [Video]. YouTube. https://youtu.be/OWZfCYze-AQ. Published November 17, 2017. |
| IO Access |  |  |
| IO placement | https://youtu.be/KHXSfh2ZRDM | Mason, J. EZ IO Placement [Video]. YouTube. <https://youtu.be/KHXSfh2ZRDM>. Published May 20, 2016. |

**SUPPLEMENTAL 3: GROUP SCENARIO DESCRIPTIONS**

**Scenario #1 - Trauma Patient**

*Scene Size Up:*

Your BLS unit arrives on scene of a MVC to find a single vehicle with impact into a telephone pole. Upon approaching the vehicle, you do not find a patient, but notice that the windshield is shattered. You look over the hillside and see a middle-aged female patient who was ejected from the vehicle. You and your partners grab your first-in bag, climb over the hillside, and approach the patient.

*Physical Assessment:*

Upon assessment, you notice that the patient is unresponsive. Skin is pale, cool, and clammy. You find a laceration to her L arm which is hemorrhaging profusely. The patient appears to be barely breathing with irregular respirations. You find major L chest trauma. Following ALS arrival, multiple attempts to place an ET tube were unsuccessful.

*Vital Signs:*

BP: 70/43, HR: 135, RR: 3, SpO2: 30%, Breath Sounds: Absent L side

*Discussion Questions:*

1. What should the BLS providers do before ALS arrival?
2. What interventions should the ALS provider perform upon arrival?
3. How can the BLS provider assist the ALS provider with these skills/interventions?
4. What exact equipment should the BLS provider gather?
5. How can the BLS provider physically assist with performing the interventions?

**Scenario #2 - Medical Patient**

*Scene Size Up:*

Your BLS unit arrives on scene of a restaurant to find a 55 y/o M patient complaining of chest pain. The patient appears to have eaten some fried food and is currently clutching his chest.

*Physical Assessment:*

Upon assessment of the patient, you see that he is profusely sweating. He complains of radiating chest pain that “just won’t go away.” You begin your BLS assessment and call for backup. Upon ALS arrival, the patient loses both radial and carotid pulses and goes into cardiac arrest. Patient developed agonal respirations upon arresting. All attempts at establishing intravenous (IV) access failed.

*Vital Signs:*

BP: 180/106 → Impalpable, HR: 168 → Absent, RR: 12 → 2 SpO2: 94% → 16%

 
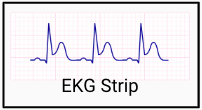


*Discussion Questions:*

1. What should the BLS providers do before ALS arrival?
2. What interventions should the ALS provider perform upon arrival?
3. How can the BLS provider assist the ALS provider with these skills/interventions?
4. What exact equipment should the BLS provider gather?
5. How can the BLS provider physically assist with performing the interventions?

**Scenario #3 - Multiple Patients**

*Scene Size Up:*

You are working a special-event where you and your EMT partners are providing coverage to a major rock concert. You are called to section 16 of the venue for “a man looking blue.” You and your partners arrive on scene to find a ~20 y/o M patient who is lodged under the seat, which is bolted to the ground. While approaching this patient, you see a ~30 y/o F collapse a few feet away and she appears to be face-down in her own vomit.

*Physical Assessment:*

Upon assessment of the male patient, you find that he is cyanotic and unresponsive. He appears to be apneic and has pinpoint pupils. Narcan was administered with no improvement. He is stuck under the seating and cannot be easily extricated. Upon assessment of the female patient, you find that she is minimally responsive. Her skin is pale and she appears to have labored respirations. She has vomit covering her face and in her mouth. Her “friend” says that she has been drinking non-stop for the entire concert.

*Vital Signs:*

20 y/o M: BP: 120/74, HR: 50, RR: 0, SpO2: - - -

30 y/o F: BP: 60/42, HR: 120, RR: 10, SpO2: 78%

*Discussion Questions:*

1. What should the BLS providers do before ALS arrival?
2. What interventions should the ALS provider perform upon arrival?
3. How can the BLS provider assist the ALS provider with these skills/interventions?
4. What exact equipment should the BLS provider gather?
5. How can the BLS provider physically assist with performing the interventions?

**SUPPLEMENTAL 4: PRE/POST-COURSE QUESTIONNAIRES**

**Pre-Course Questionnaire**

This is a voluntary research study in which you will complete a survey. This will help us determine the efficacy of a continuing education course in preparing BLS providers to assist with ALS interventions. The survey will be anonymous as we will not be collecting any information that can identify you. Choosing not to participate will not affect your status or participation within this course, however, contributing will help advance the field of EMS education. The time to complete this will be approximately 10 minutes. If you have any questions, you can contact the study co-principal investigator, Enzo Plaitano, at 724-961-1099 or enzop@bu.edu. This study was approved by the Boston University Institutional Review Board (H-40458).

1. Yes, I consent to participate
2. No, I do not consent to participate

**PAGE 1 - Demographics Information**

- - 1. Approximately how many years have you been an EMT (BLS Provider)?

__________________

- - 1. To which gender do you most identify?

Woman

Man

Non-binary/Gender non-conforming

Prefer to self-describe

Prefer not to say

1. Do you currently or have you consistently worked as part of an ALS unit?

Yes

No

1. While working alongside an ALS provider, how often do you assist that ALS provider with performing ALS interventions?

Never

Rarely

Sometimes

Often

Always

1. Have you ever attended another formal class discussing BLS-ALS interface and how to better assist the ALS provider?

Yes

No

1. Do you believe that conventional EMT class education adequately prepared you to work with ALS providers and assist with ALS interventions?

Yes

No

1. Did you feel comfortable working as part of an ALS unit upon completion of conventional EMT class?

Yes

No

**PAGE 2 - Qualitative Survey**

1. How comfortable do you feel communicating with ALS providers and voicing your opinion regarding patient care?

Very uncomfortable

Uncomfortable

Neutral

Comfortable

Very comfortable

1. How comfortable do you feel with EKG equipment and assisting ALS providers with performing EKGs?

Very uncomfortable

Uncomfortable

Neutral

Comfortable

Very comfortable

1. How comfortable do you feel with advanced airway equipment and assisting ALS providers with performing advanced airways?

Very uncomfortable

Uncomfortable

Neutral

Comfortable

Very comfortable

1. How comfortable do you feel with needle decompression equipment and assisting ALS providers with performing chest needle decompression?

Very uncomfortable

Uncomfortable

Neutral

Comfortable

Very comfortable

1. How comfortable do you feel with intravenous (IV) equipment and assisting ALS providers with establishing intravenous (IV) access?

Very uncomfortable

Uncomfortable

Neutral

Comfortable

Very comfortable

1. Overall, how comfortable do you feel setting-up ALS equipment and assisting with ALS interventions?

Very uncomfortable

Uncomfortable

Neutral

Comfortable

Very comfortable

**PAGE 3 - Cognitive Quiz**

1. Your unit is establishing IV access in a 30 y/o patient and you overhear the paramedic say, “she has good venous access in the left arm.” While preparing the supplies for the ALS provider, what size catheter will you grab?
   1. 24GA
   2. **20GA**
   3. 16GA
   4. 14GA
2. Your unit is establishing IV access in a patient and you are preparing and priming the saline bag for connection to the catheter. There are several packages of primary tubing, all with different flow rates. Which of the following will you grab?
   1. **15 drops/mL**
   2. 30 drops/mL
   3. 45 drops/mL
   4. 75 drops/mL
3. Your unit is establishing IV access in a patient with dehydration. The paramedic asks you to gather the supplies. Which of the following will you NOT hand to the paramedic?
   1. Appropriately sized IV catheter
   2. Alcohol swab
   3. Extension set “Loop”
   4. **Empty 10mL syringe**
4. Your unit is working a cardiac arrest and the paramedic asks you to prepare the intraosseous (IO) drill to establish left humeral access. Which needle will you apply to the end of the drill before handing it to the paramedic?
   1. Pink (15mm)
   2. Blue (25mm)
   3. **Yellow (45mm)**
   4. Purple (75mm)
5. Your unit is treating a patient complaining of chest pain. The paramedic asks you to apply the electrodes for a 12-lead EKG. How many electrodes will you be placing on the entire patient?
   1. 6
   2. 8
   3. **10**
   4. 12
6. Your unit is treating a patient complaining of chest pain. The paramedic applied a 12-lead EKG, but you notice that lead **V2 (yellow)** has fallen off and is now hanging from the patient. The paramedic is busy dosing cardiac medications, so you grab the electrode/lead and reapply it to which correct location on the chest?
   1. Right chest near the clavicle bone
   2. **4th intercostal space, left sternal edge**
   3. 5th intercostal space, mid-clavicular line
   4. Mid-axillary line, in line with V4 and V5
7. Your unit is treating a patient with chest trauma and decreased lung sounds. The new paramedic is frazzled and turns to you to ask where to insert the needle for the chest decompression. What site do you tell them?
   1. **2nd intercostal space, mid-clavicular line**
   2. 4th intercostal space, sternal edge
   3. 5th intercostal space, mid-clavicular line
   4. 6th intercostal space, mid-axillary line
8. Your unit is preparing to intubate an unresponsive adult patient of normal size. The paramedic asks you to prepare the equipment and says “choose whatever blade you want, I don’t care.” Based on the highest success rates and appropriate sizing, which blade would you choose for the best use in this adult patient.
   1. Miller, Size 3
   2. Miller, Size 5
   3. **Macintosh, Size 3**
   4. Macintosh, Size 5
9. Your unit is treating a patient with an uncontrolled airway. The paramedic asks you to prepare equipment to establish the most definitive airway. Which airway device do you select for this patient?
   1. Laryngeal Mask Airway
   2. King Airway
   3. **Endotracheal Tube**
   4. Oropharyngeal Airway
10. This EKG shows which rhythm?


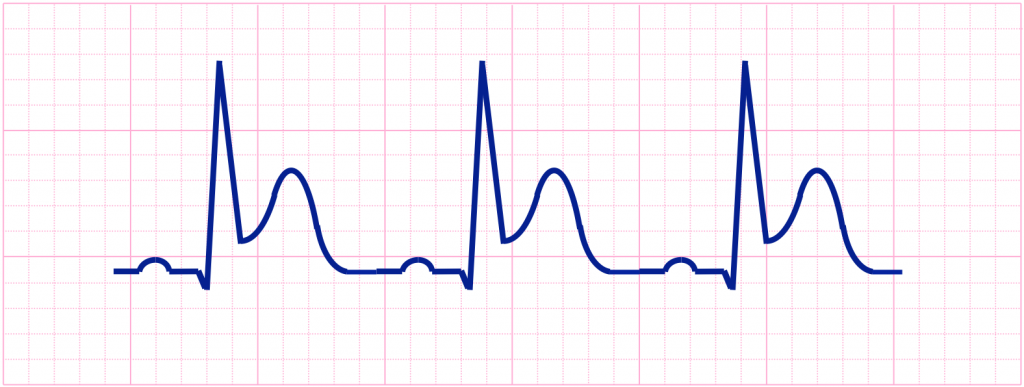


- 1. Atrial flutter
  2. Normal sinus rhythm
  3. **STEMI**
  4. Ventricular tachycardia

- - - - - - - - - - - - - - - - - - - - -- - - - - - - - - - - - - - - - - - - - - - - - - - - - - -

**Post-Course Questionnaire**

This is a voluntary research study in which you will complete a survey. This will help us determine the efficacy of a continuing education course in preparing BLS providers to assist with ALS interventions. The survey will be anonymous as we will not be collecting any information that can identify you. Choosing not to participate will not affect your status or participation within this course, however, contributing will help advance the field of EMS education. The time to complete this will be approximately 10 minutes. If you have any questions, you can contact the study co-principal investigator, Enzo Plaitano, at 724-961-1099 or enzop@bu.edu. This study was approved by the Boston University Institutional Review Board (H-40458).

1. Yes, I consent to participate
2. No, I do not consent to participate

**PAGE 1 - Qualitative Survey**

1. The breakout room and debriefing component of this course was valuable:

Strongly disagree

Disagree

Neutral

Agree

Strongly agree

1. The breakout rooms and debriefing component allowed me to better understand the multidisciplinary (BLS-ALS ) care of complex medical and trauma patients:

Strongly disagree

Disagree

Neutral

Agree

Strongly agree

1. The breakout rooms and debriefing component allowed me to better conceptualize which interventions the ALS provider may perform for simulated patient scenarios:

Strongly disagree

Disagree

Neutral

Agree

Strongly agree

1. The lecture component of this course was valuable:

Strongly disagree

Disagree

Neutral

Agree

Strongly agree

1. The lecture component allowed me to better understand ALS equipment and the steps in preparing appropriate supplies for ALS interventions:

Strongly disagree

Disagree

Neutral

Agree

Strongly agree

1. The video component of this course was valuable:

Strongly disagree

Disagree

Neutral

Agree

Strongly agree

1. After this course, I am more likely to assist ALS providers in setting-up equipment and assisting with interventions:

Strongly disagree

Disagree

Neutral

Agree

Strongly agree

1. After this course, how comfortable do you feel communicating with ALS providers and voicing your opinion regarding the care of an ALS patient?

Very uncomfortable

Uncomfortable

Neutral

Comfortable

Very comfortable

1. After this course, how comfortable do you feel with EKG equipment and assisting ALS providers with performing EKGs?

Very uncomfortable

Uncomfortable

Neutral

Comfortable

Very comfortable

1. After this course, how comfortable do you feel with advanced airway equipment and assisting ALS providers with performing advanced airways?

Very uncomfortable

Uncomfortable

Neutral

Comfortable

Very comfortable

1. After this course, how comfortable do you feel with needle decompression equipment and assisting ALS providers with performing chest needle decompressions?

Very uncomfortable

Uncomfortable

Neutral

Comfortable

Very comfortable

1. After this course, how comfortable do you feel with intravenous (IV) equipment and assisting ALS providers with establishing IV access?

Very uncomfortable

Uncomfortable

Neutral

Comfortable

Very comfortable

1. Overall, after this course, how comfortable do you feel setting-up ALS equipment and assisting with ALS interventions?

Very uncomfortable

Uncomfortable

Neutral

Comfortable

Very comfortable

**PAGE 2 - Cognitive Quiz**

1. Your unit is establishing IV access in a 30 y/o patient and you overhear the paramedic say, “she has good venous access in the left arm.” While preparing the supplies for the ALS provider, what size catheter will you grab?
   1. 24GA
   2. **20GA**
   3. 16GA
   4. 14GA
2. Your unit is establishing IV access in a patient and you are preparing and priming the saline bag for connection to the catheter. There are several packages of primary tubing, all with different flow rates. Which of the following will you grab?
   1. **15 drops/mL**
   2. 30 drops/mL
   3. 45 drops/mL
   4. 75 drops/mL
3. Your unit is establishing IV access in a patient with dehydration. The paramedic asks you to gather the supplies. Which of the following will you NOT hand to the paramedic?
   1. Appropriately sized IV catheter
   2. Alcohol swab
   3. Extension set “Loop”
   4. **Empty 10mL syringe**
4. Your unit is working a cardiac arrest and the paramedic asks you to prepare the intraosseous (IO) drill to establish left humeral access. Which needle will you apply to the end of the drill before handing it to the paramedic?
   1. Pink (15mm)
   2. Blue (25mm)
   3. **Yellow (45mm)**
   4. Purple (75mm)
5. Your unit is treating a patient complaining of chest pain. The paramedic asks you to apply the electrodes for a 12-lead EKG. How many electrodes will you be placing on the entire patient?
   1. 6
   2. 8
   3. **10**
   4. 12
6. Your unit is treating a patient complaining of chest pain. The paramedic applied a 12-lead EKG, but you notice that lead **V2 (yellow)** has fallen off and is now hanging from the patient. The paramedic is busy dosing cardiac medications, so you grab the electrode/lead and reapply it to which correct location on the chest?
   1. Right chest near the clavicle bone
   2. **4th intercostal space, left sternal edge**
   3. 5th intercostal space, mid-clavicular line
   4. Mid-axillary line, in line with V4 and V5
7. Your unit is treating a patient with chest trauma and decreased lung sounds. The new paramedic is frazzled and turns to you to ask where to insert the needle for the chest decompression. What site do you tell them?
   1. **2nd intercostal space, mid-clavicular line**
   2. 4th intercostal space, sternal edge
   3. 5th intercostal space, mid-clavicular line
   4. 6th intercostal space, mid-axillary line
8. Your unit is preparing to intubate an unresponsive adult patient of normal size. The paramedic asks you to prepare the equipment and says “choose whatever blade you want, I don’t care.” Based on the highest success rates and appropriate sizing, which blade would you choose for the best use in this adult patient.
   1. Miller, Size 3
   2. Miller, Size 5
   3. **Macintosh, Size 3**
   4. Macintosh, Size 5
9. Your unit is treating a patient with an uncontrolled airway. The paramedic asks you to prepare equipment to establish the most definitive airway. Which airway device do you select for this patient?
   1. Laryngeal Mask Airway
   2. King Airway
   3. **Endotracheal Tube**
   4. Oropharyngeal Airway
10. This EKG shows which rhythm?


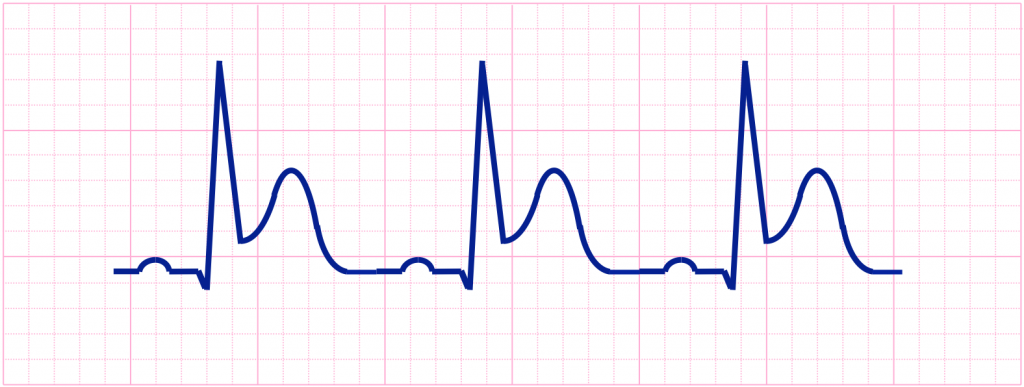


- 1. Atrial flutter
  2. Normal sinus rhythm
  3. **STEMI**
  4. Ventricular tachycardia
